# The role of SDF_1α/CXCR4 axis in non-antibody mediated TRALI in cardiac surgery patients

**DOI:** 10.1186/s40635-026-00919-z

**Published:** 2026-07-23

**Authors:** Mickael Vourc’h, Enora Ferron, Cécile Poulain, Gaëlle David, Christelle Volteau, Jérémie Poschmann, Martin Braud, Regis Josien, Bertrand Rozec, Jose A Villadangos, Christelle Retiere, Karim Asehnoune

**Affiliations:** 1 Department of Microbiology and Immunology, The Peter Doherty Institute for Infection and Immunity, The University of Melbourne, Parkville, VIC 3010 Australia; 2 INSERM Center for Research in Transplantation and Translational Immunology, UMR 1064, Nantes Université, Nantes, France; 3 Cardiac Surgery Intensive Care Unit, Hôpital Laennec, University Hospital of Nantes, 44000 Nantes, France; 4 Etablissement Français du Sang (EFS) Pays-de-Loire, Nantes, France; 5 Team 12 CRCI²NA, INSERM UMR1307, CNRS UMR 6075, Université de Nantes, Nantes, France; 6 Surgical Intensive Care Unit, Hôtel Dieu, University Hospital of Nantes, 44000 Nantes, France; 7 Plateforme de Méthodologie et Biostatistique, Université de Nantes, Nantes, France; 8 Nantes Université, CHU Nantes, Laboratory of Immunology, CIMNA, Nantes, France; 9 CNRS, INSERM, l’institut du Thorax, Nantes Université, 44093 Nantes, France; 10 Department of Biochemistry and Pharmacology, The University of Melbourne, Bio21 Molecular Science and Biotechnology Institute, Parkville, VIC 3010, Australia

**Keywords:** Cardiac surgery, Transfusion, Red blood cell, Respiratory failure, CXCL12, SDF_1α

## Abstract

**Background:**

Acute lung injury (ALI) after extracorporeal circulation can originate from multiple causes, including transfusion. Despite improvements in blood safety policy, transfusion-related lung injury (TRALI) continues to occur, suggesting an incomplete understanding of its pathogenesis.

**Objectives:**

To evaluate the association between the composition of the red blood cell unit (RBC) and the onset of ALI after transfusion, defined by a ratio between partial pressure of oxygen (PaO_2_) and the fraction of inspired oxygen (FiO_2_) ≤ 300 mmHg in the first 3 days postoperatively.

**Methods:**

Adults undergoing scheduled cardiac surgery at Nantes University Hospital between September 2016 and March 2021 and requiring transfusion of 1–5 RBC during surgery were included. To determine the exposure of each patient (i.e., total amount of inflammatory proteins received during transfusion), we analyzed the composition (panel of 15 proteins) of each RBC received by the participants. After stimulation of peripheral blood mononuclear cells (PBMC) with supernatant RBC (SN-RBC), NK cell cytotoxicity to pulmonary epithelial cells (Calu_3 cells) was assessed. Finally, we determined the association between SN-RBC composition, patient characteristics, and blood donation preparation methods.

**Results:**

Over the study period, 161 patients were included, of whom 54 (33.5%) developed ALI within the first 3 days. Patients with ALI had a significantly higher median (IQR) exposure to SDF_1α than non-ALI patients 2.0 × 10^4^ (0.0 to 4.2 × 10^4^) vs. 1.0 × 10^4^ (0.0 to 2.7 × 10^4^) picograms, *P* = 0.02. In vitro, PBMC coculture with SN-RBC containing high levels of SDF_1α enhanced NKG2D-dependant NKG2A^+^ NK cell cytotoxicity to Calu_3 cells. RBC from female donors or donors with platelet counts higher than 300 × 10^9^/L showed the highest concentration of SDF_1α, as well as RBC prepared by "whole blood filtration".

**Conclusions:**

High exposure to SDF_1α through transfusion may be associated with the onset of ALI following cardiac surgery. The increase in NKG2D-dependent cytotoxicity of NKG2A^+^ NK cells is a possible explanation for this finding. These results advocate for better characterization of the determinants of RBC composition and for developing new immune strategies to mitigate transfusion side effects.

**Supplementary Information:**

The online version contains supplementary material available at 10.1186/s40635-026-00919-z.

## Introduction

Approximately 1.5 million patients undergo cardiac surgery worldwide each year [1]. Overall mortality is estimated at 2%-5% [2] and morbidity including vital organ dysfunction can reach 70% [3]. Acute lung injury (ALI) is a frequent (5% to 20%) and life-threatening condition after extracorporeal circulation [4, 5]. It increases time on ventilator, duration of stay in the ICU, and mortality [6]. ALI often originates from multiple intricated factors such as pleural effusion, atelectasis, cardiogenic pulmonary edema, ischemia–reperfusion and transfusion [7, 8].

During cardiac surgery, 15%-30% of patients receive red blood cell transfusions in order to overcome blood loss. Nevertheless, each additional transfusion of red blood cell units (RBC) can increase postoperative morbidity and mortality [9, 10]. Regarding lung dysfunction after transfusion [11], two entities are usually described: transfusion-associated circulatory overload (TACO) and transfusion-related acute lung injury (TRALI). The latter corresponds to an acute onset of respiratory failure after transfusion, defined as hypoxemia (PaO_2_/FiO_2_ ratio ≤ 300 mmHg or SpO_2_ < 90% in room air) with bilateral infiltrate on chest X-ray, and without evidence of left atrium hypertension. TRALI is potentially fatal owing to the lack of effective strategies and has long been the leading cause of transfusion-related mortality prior to the improvement of blood safety policy. However, cases continue to occur, highlighting an incomplete understanding of its pathogenesis. Currently, TRALI is described as a two-hit model [12–14]. The first hit is related to the patient’s condition (i.e., severe burn injury, active infection, ischemia–reperfusion during surgery, or severe trauma), which triggers a generalized inflammatory response. In the lung compartment, it leads to the activation of pulmonary endothelium and adhesion of primed leukocytes including neutrophils, monocytes or natural killer (NK) cells [15]. The second hit is the administration of blood product containing either antibodies (targeting human leukocyte antigens or human neutrophil antigens) [14], or non-antibody proinflammatory mediators [16]. It triggers the transmigration of the primed leukocytes into the lungs and enhances their cytotoxic function to endothelium or epithelium [16].

Cardiopulmonary bypass (CPB), which is mandatory to ensure organ oxygenation during open-heart surgery, is often described as an experimental model of ischemia–reperfusion and stands as a "first hit". This damages the kidneys, lungs, brain, or heart and generates a local and general inflammatory response. Our team previously demonstrated that components of RBC can act as the "second hit" and lead to kidney damage [17]. We therefore investigated whether this was transposable to lung damage. The roles of neutrophils [18] and monocytes in TRALI have been previously reported [19], but little is known about the role of NK cells. We hypothesized that specific components of RBC could modulate NK-mediated ALI in the context of transfusion in patients undergoing cardiac surgery.

## Materials and methods

### Study setting and ethics statement

The TRANSNEPHRON study was a monocentric prospective study. Patients were recruited from the Nantes University Hospital between September 2016 and March 2021. The study was registered at clinicaltrial.gov (NCT02763410) before the first inclusion. The protocol was reviewed by an institutional board and the participants received oral and written information during the preoperative consultation. Patients provided written consent for the storage of biological samples (Ethics Committee Ouest V, Rennes, #16/02–1000, approval March 4, 2016).

### Participants, study design, and collection of biological samples

**Fig. 1:**
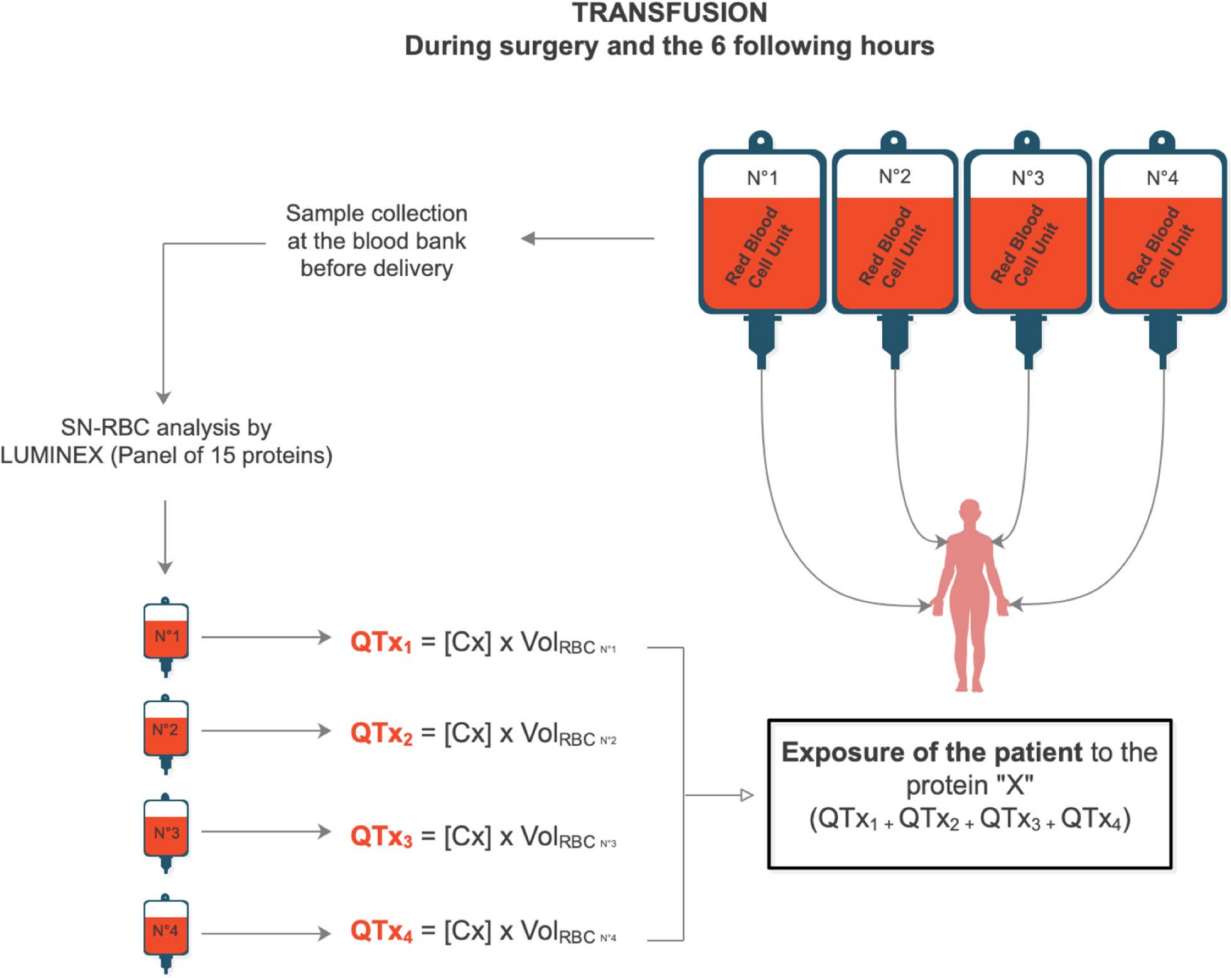
Experimental design and exposure definition. All participants underwent extracorporeal circulation (ECC) to ensure organ oxygenation during surgery. ECC bypasses the heart by draining the right atrium and reinjecting continuous blood into the ascending aorta. Although ECC is a well-established strategy to ensure patient safety during surgery, the reduction of blood flow by up to 30% compared with physiological conditions, as well as the modification of the perfusion regimens, are responsible for ischemia–reperfusion of vital organs such as the kidneys and lungs. During cardiac surgery and the following 6 h, a sample of each Red Blood Cell (RBC) unit received by the patients was collected. The total amount of each protein (i.e., QT_x_ for protein “X”) was determined in each RBC by multiplying its concentration [C_x_] by the volume of each RBC. If a patient received 3 RBCs transfusions during surgery, exposure to “X” was defined as QT_x1_ + QT_x2_ + QT_x3_

Patients undergoing scheduled cardiac surgery with cardiopulmonary bypass were eligible if they received 1–5 RBC units during surgery or the six hours following surgery. Non-inclusion criteria were as follows: transfusion of any blood product before surgery or within the previous 90 days, heart and/or lung transplantation surgery, emergency surgery to be performed within 24 h, age < 18 years, pregnancy, protected adults or incapable of expressing their non-opposition, opposition expressed by the patient on recording his/her data, no French health insurance, endocarditis, myocardial infarction in the past 15 days, and hemodynamic instability requiring inotropic or vasopressor drugs before surgery. Patients who received a transfusion of fresh frozen plasma or platelet concentrate during surgery and the 6 following hours were excluded. A sample of each RBC transfused to the participants was drawn by the transfusion center and stored for long-term conservation (see Fig. 1).

### Objectives and endpoints

The primary objective was to determine whether the exposure of the participants during surgery or in the following 6 h was associated with the onset of ALI (i.e., defined as a PaO_2_/FiO_2_ ratio ≤ 300 mmHg at least once in the first three days after cardiac surgery). To determine patient exposure, the composition of the RBC supernatant (SN-RBC) was analyzed using a multiplex immune-assay for a panel of 15 proteins. Briefly, exposure (e.g., to protein "X") was defined as the total amount of "X" received during transfusion. It was determined by adding the quantity of "X" in each of the SN-RBC transfused to the patient (Fig. 1). We then compared the exposure of patients with ALI and non-ALI patients. The results of the primary analysis were transposed in vitro to a co-culture model of human peripheral blood mononuclear cells (PBMC) to assess the effect of SN-RBC composition on NK cell cytotoxicity to the Calu_3 lung epithelial cell line. Finally, we analyzed whether the composition of the RBC was related to donor characteristics and/or preparation methods. After the recruitment of 105 patients (in September 2018), part of this cohort was analyzed focusing on the effects of transfusion on acute kidney injury after cardiac surgery [17].

### Multiplex immune-assay

In order to determine the inflammatory contents of each SN-RBC, the following kits were used for human multiplex immune-assay: HMGB1 and HSP_70 (Merck Millipore, Molsheim, France), MRP_14 (Bio-Techne SAS, Rennes)**.** All other human kits were purchased from Life Technologies, Courtaboeuf. The plates were analyzed using a MagPix instrument (Luminex Corp).

### PBMC from healthy donors, Calu_3 and HK_2 cell lines

PBMC were isolated from heparinized blood by gradient centrifugation on Ficoll-Hypaque and frozen (Lymphoprep, Norway) from three healthy volunteer blood donors (Etablissement Français du Sang, Nantes, France).

Calu_3 cells (EFS-PL laboratory, Nantes, France) are lung epithelial cells isolated from non-small-cell lung adenocarcinoma that grow in adherent cultures. This cell line was cultured at 37 °C in 5% CO_2_ in ATCC-formulated Eagle's Minimum Essential Medium, Catalog No. 30-2003 supplemented with 10% Fetal Calf Serum. HK_2 cells (from EFS-PL laboratory, Nantes, France) are proximal tubular cell lines derived from human kidneys cultured at 37 °C in 5% CO_2_ in keratinocyte serum-free medium. See Supplemental eTable 1 for full HLA phenotyping of Calu_3 and HK_2 cell lines. Calu_3 and HK_2 cells were used as "target" to assess NK cell cytotoxicity.

### Co-culture model: PBMC stimulation with SN-RBC and NK cell cytotoxicity assay (CD107a mobilization assay)

Before stimulation, PBMCs were cultured at 37 °C in 5% CO_2_ overnight in RPMI 1640 medium (Gibco) containing glutamine (Gibco) with 10% fetal bovine serum (Gibco; 10 endotoxin units (EU)/ml endotoxin contamination), and supplemented with 100 U/ml IL_2 (100 U/ml Proleukin [aldesleukin]; Chiron). PBMC were seeded in a 96-well plate (250,000 per well) and stimulated for 30 min with SN-RBC (immediately after unfreezing) or medium (unstimulated condition) without IL_2. Then, 1 μL of anti-CD107a-BV421 was added to each well and Calu_3/or HK_2 targets were added to the appropriate wells (PBMC: target ratio, 10:1). Brefeldin A was added 1 h later (final concentration 5 μg/mL) and co-culture was continued 4 h before cytometry analysis.

The CD107a mobilization assay was reproduced with 12 distinct SN-RBC and each SN-RBC was tested with three distinct healthy donors. The results are expressed as the mean cytotoxic activity of the three distinct donors. Statistical analysis of the cytotoxicity assay was conducted blindly from the results of the Luminex immune-assay which determined the SN-RBC composition.

### NKG2D blocking

The anti-hNKG2D antibody (IgG1, clone 149,810; R&D) or its isotype (IgG1, clone NCG01; Invitrogen) were incubated with sorted NK cells (untouched NK cell isolation kit) for 1 h (4°C) in 100 μL of IL_2 medium with PS for final concentrations of 75 and 37.5 μg/ml. The cytotoxic assay was subsequently performed as described above with the Calu_3 cell line (ratio NK:Calu_3 of 1:1).

### Cell labeling

Antibodies were purchased from BD Biosciences, unless otherwise stated. Data were collected using a 4-color FACSCalibur (BD Biosciences) and LSR II cytometer (Beckton Dickinson, Le Pont de Claix, France) and analyzed using FlowJo 6.2 software (FlowJo, LLC, Ashland, OR, USA). For PBMCs, NK cell gating was performed using anti-CD56-BV510 (HCD56, Ref. #2,191,695), anti-CD3-PerCP (SK7; Ref. #2,324,090) and the corresponding isotype-matched control monoclonal antibody (MAb). NK cell receptor phenotyping was performed with anti-NKp30-PE (Z25, Beckman Coulter, Ref. #IM3709), anti-NKp44-PE-Cy7 (P44-8, Ref. #325,116), anti-NKp46-APC (9E25, Ref. #2,259,590), anti-NKG2D-PerCP-Cy5.5 (1D11, Ref. #2,204,090), anti-KIR_2DL1,_2DS1-FITC (11PB6/EB6, Miltenyi Biotec, Ref. #130-092-811), anti-NKG2C-PE (134,591, R&D systems, Ref. #FAB138P), anti-NKG2A-PC7 (Z199, Beckman Coulter, Ref. #B10246), anti-KIR_2DL2,_2DS2,_2DL3-APC (GL183, Beckman Coulter, Ref. #A22333), anti-DNAM1-PerCP-Cy5.5 (11A8, Ref. #339,314) mAbs. The expression of the ligands of NK cells activating receptors on Calu_3 cells was determined using anti-ULBP1 (170,818), anti-ULBP 2, 5, 6 (165,903), anti-ULBP 3 (166,510), anti-ULBP 4 (709,116), anti-B7-H6 (8,750,001; R&D Systems, Minneapolis, MN, USA), anti-MICA/B (6D4), anti-CD112 (R2.525, BD Biosciences, Le Pont de Claix, France), anti-CD48 (BJ40), and anti-CD155 (SKII.4; BioLegend, San Diego, CA, USA). For the mobilization assay, cells were stained during the 4-h exposition to Calu_3 cells with an anti-CD107a-BV421 MAb (clone H4A3, Ref. #2,243,130). Viability was assessed using the APC fixable viability dye kit eFluor 780 staining (eBioscience).

### Statistical analysis

The number of subjects to be enrolled in the Transnephron study was not calculated according to standard methods given the lack of data available on the association between RBC unit composition and the onset of ALI after cardiac surgery. This calculation was therefore based on the feasibility of the project in the Cardiac Surgery Department of Nantes University Hospital. Analyses were performed using GraphPad Prism software (La Jolla, CA, USA).

Baseline characteristics are reported as numbers (percentages) for qualitative variables and as means (standard deviations) or medians (interquartile ranges) according to distribution for quantitative variables.

The Kruskal–Wallis test was used to compare multiple groups. Post-hoc Dunn’s test was used to perform multiple comparisons. Mann–Whitney tests were used to compare exposures of patients with ALI (PaO_2_/FiO_2_ ≤ 300 mmHg group) and non-ALI patients (PaO_2_/FiO_2_ > 300 mmHg group), with a *P*-value of < 0.05 considered to be statistically significant (primary outcome).

### Differential gene expression

To constitute the cohort for RNA sequencing, PBMCs from 42 healthy volonteers were unfrozen the day before cell sorting and cultured overnight in RPMI 1640 medium containing glutamine and penicillin–streptomycin and supplemented with 10% FBS (Gibco). NK cells were stained with labelled mAbs specific for CD3, CD56, NKp46, NKG2A. The stained cells were sorted into two subsets NKG2A^−^ and NKG2A^+^ using a FACSAria™ III cell sorter (BD Biosciences). Total RNA was extracted directly, quantified and 3’ digital gene expression profiling was carried out [20]. Raw sequencing data were deposited in GEO under accession number (GEO GSE281383). Data were analysed using the 3’ Sequencing RNA Profiling (SRP) pipeline [21]. Conditions were compared using the DESeq2 R package. This methodology was derived from previous publications [20, 22]. The complete material and method for the bioinformatics part is available in Supplemental #1.

## Results

### Baseline characteristics of the population

**Fig. 2:**
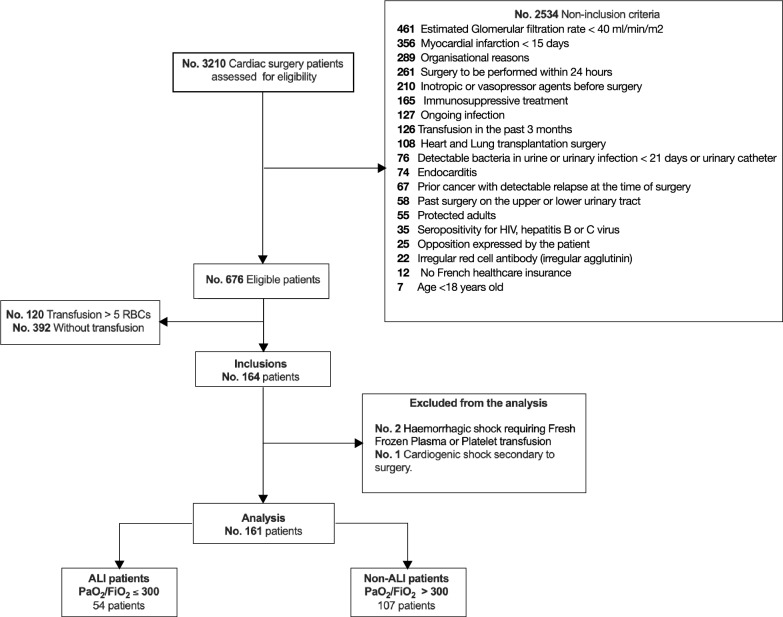
Flow Chart. This observational study was conducted in the cardiac surgery intensive care unit of Nantes University Hospital between September 2016 and March 2021. ALI, acute lung injury; RBC, red blood call units

Over the study period, 164 patients were included between September 2016 and March 2021. Three patients were excluded from the analysis owing to hemorrhagic shock (n = 2) or cardiogenic shock after surgery. Finally, 161 patients were analyzed with 54 patients (33.5%) presenting with ALI and 107 patients (66.5%) non-ALI patients (Fig. 2). The characteristics of the population are shown in Table 1. Patients with ALI had a significantly higher mean BMI (28.1 ± 5.1 vs. 24.9 ± 4.4, *P* < 0.01) and higher mean theoretical cardiac output (4.50 ± 0.5 vs. 4.10 ± 0.5, *P* < 0.01) than non-ALI patients. There was no difference between the ALI and non-ALI patients in the median [IQR] number of RBC transfused intraoperatively and in the following 48 h: 2 [1.0–2.0] vs. 2 [1.0–2.0], *P* = 0.75. Patients with ALI consistently spent longer mean time on the ventilator than non-ALI patients: 52.5 (± 83.3) hours vs. 18.2 (± 61.4) hours, *P* = 0.03.

**Table 1:** Population characteristics

	ALI patients PaO_2_/FiO_2_ ratio ≤ 300 N = 54	Non-ALI patients PaO_2_/FiO_2_ ratio > 300 N = 107	*P*-value
Sex, male, no (%)	26 (48.2%)	36 (33.6%)	0.07
Age, mean (± SD), years	71.4 (± 11.2)	72.2 (± 7.9)	0.64
BMI, mean (± SD)	28.1 (± 5.1)	24.8 (± 4.4)	< 0.01
Theoric cardiac output, mean (± SD), L/min/m^2^	4.4 (± 0.5)	4.1 (± 0.5)	< 0.01
Active tobacco, n° (%)	13 (24.1%)	30 (28.0%)	0.59
Diabetes, n° (%)	15 (27.8%)	22 (20.6%)	0.30
Medical history of cancer, n° (%)	10 (18.5%)	19 (17.8%)	0.91
Preoperative biology			
Hemoglobin, mean (± SD), g/dL	12.4 (± 1.5)	12.2 (± 1.4)	0.45
Platelets, mean (± SD), × 10^9^/L	254.0 (± 88.1)	247.2 (± 68.8)	0.62
Quick Time, mean (± SD), %	88.6 (± 18.1)	90.8 (± 16.8)	0.44
Creatinine, mean (± SD), µmol/L	87.8 (± 25.9)	81.0 (± 19.5)	0.09
Surgery, no (%)			
Coronary artery bypass graft	17 (31.5%)	28 (26.2%)	0.08
Aortic or mitral valve replacement	8 (14.8%)	35 (32.7%)	
Combined surgery	29 (53.7%)	44 (41.1%)	
Mean duration of extracorporeal circulation, minutes (± SD)	124.9 (± 43.9)	113.2 (± 43.6)	0.11
Mean aortic cross-clamping, minutes (± SD)	100.0 (± 39.4)	86.2 (± 35.7)	0.03
Transfusion median [IQR]			
No. of RBC during surgery and the following 6 h	2.0 [1.0 to 2.0]	2.0 [1.0 to 2.0]	0.75
Transfusion after the 6th hour postoperatively			
No. of RBC	4.0 [2.0 to 7.0]	3.0 [2.0 to 3.0]	0.14
No. of plasma	5.0 [3.0 to 7.0]	4.0 [2.0 to 7.0]	0.71
No. of platelet concentrate	1.0 [1.0 to 3.0]	2.0 [2.0 to 3.0]	0.47
Postoperative outcomes			
Highest level of arterial lactate, mean (± SD), mmol/L	2.2 (± 1.8)	2.0 (± 2.1)	0.56
PaO_2_/FiO_2_ ratio at day 1, mean (± SD), mmHg	179.1 (± 56.7)	333.6 (± 94.4)	< 0.01
Time on ventilator, mean (± SD), hours	52.5 (± 83.3)	18.2 (± 61.4)	0.03
Pneumonia in the first 28 days, n° (%)	6 (11.1%)	5 (4.7%)	0.18
Deaths, n° (%)	3 (5.6%)	3 (2.8%)	0.40

*SD* Standard deviation, *PaO*_*2*_ partial pressure of arterial oxygen, *FiO*_*2*_ fraction of inspired oxygen, *BMI* Body Mass Index, *RBC* red blood cells, *IQR* interquartile range

### Patient exposure and ALI onset

In order to determine patient exposure to inflammatory proteins in blood products, the composition of the 204 RBC transfused to the 161 patients was analyzed. ALI patients had greater median (IQR) exposure to SDF_1α and MRP_14 than non-ALI patients, respectively, 2.0 × 10^4^ (0.0 to 4.2 × 10^4^) vs. 1.0 × 10^4^ (0.0 to 2.7 × 10^4^) picograms, *P* = 0.02 and 4.9 × 10^4^ (2.9 × 10^4^ to 7.9 × 10^4^) vs. 3.2 × 10^4^ (1.7 × 10^4^ to 5.8 × 10^4^), *P* = 0.03 (Table 2**)**. Interestingly, high MRP_14 exposure has been previously described as a risk factor for transfusion-related acute kidney injury after cardiac surgery [17]. There was no difference in exposure between the groups for other proteins. Patients with the highest exposure to SDF_1α (i.e., > 50th percentile exposure of all participants) also presented more frequently with PaO_2_/FiO_2_ ≤ 200 mmHg (*P* < 0.01) and acute kidney injury (*P* < 0.01) postoperatively without difference in the duration of mechanical ventilation (Supplemental eTable 2, post hoc analysis). To determine whether these results corresponded to a relevant biological effect, we transposed our findings to an in vitro co-culture model. Specifically, we investigated whether SDF_1α and MRP_14 could be candidates for the role of "second hit" and participate in non-antibody-mediated TRALI.

**Table 2:** Exposure of patients according to the worst PaO_2_/FiO_2_ ratio in the first 5 days

Quantity of protein transfused (pg), Median (IQR) [Min–Max]	ALI patients PaO_2_/FiO_2_ ratio ≤ 300 N = 54	Non-ALI patients PaO_2_/FiO_2_ ratio > 300 N = 107	*P*
CD28	1.2 × 10^4^ (0.3 × 10^4^ to 2.6 × 10^4^) [0.0—13.7 × 10^4^]	1.0 × 10^4^ (0.0 to 2.4 × 10^4^) [0.0—11.0 × 10^4^]	0.23
CD40	4.0 × 10^2^ (0.0 to 16.7 × 10^2^) [0.0—94.0 × 10^2^]	4.0 × 10^2^ (0.0 to 23.9 × 10^2^) [0.0—97.9 × 10^2^]	0.70
FAS_Ligand	2.5 × 10^4^ (0.0 to 5.3 × 10^4^) [0.0—36.8 × 10^4^]	2.0 × 10^4^ (0.0 to 6.4 × 10^4^) [0.0—176.5 × 10^4^]	0.87
HMGB_1	6.9 × 10^7^ (3.6 × 10^7^ to 21.3 × 10^7^ [0.0—339.5 × 10^7^]	5.2 × 10^7^ (2.6 × 10^7^ to 18.7 × 10^7^) [0.0—927.0 × 10^7^]	0.97
HSP_70	2.8 × 10^7^ (1.5 × 10^7^ to 4.8 × 10^7^ [0.6 × 10^7^–18.1 × 10^7^]	2.6 × 10^7^ (1.6 × 10^7^ to 4.5 × 10^7^) [0.3 × 10^7^—15.2 × 10^7^]	0.71
IDO	8.8 × 10^3^ (0.0 to 17.9 × 10^3^) [0.0—57.8 × 10^3^]	7.7 × 10^3^ (0.0 to 15.2 × 10^3^) [0.0—52.1 × 10^3^]	0.94
IL_21	0.0 (0.0 to 1.1 × 10^4^) [0.0—3.8 × 10^4^]	0.0 (0.0 to 0.1 × 10^4^) [0.0—2.3 × 10^4^]	0.10
PD-L2	1.5 × 10^5^ (0.7 × 10^5^ to 2.2 × 10^5^) [0.0—10.3 × 10^5^]	1.4 × 10^5^ (0.8 × 10^4^ to 2.3 × 10^5^) [0.0—5.9 × 10^5^]	0.85
RANTES	2.5 × 10^4^ (1.2 × 10^4^ to 3.9 × 10^4^) [0.0—10.6 × 10^4^]	2.0 × 10^4^ (0.6 × 10^4^ to 3.6 × 10^4^) [0.0—13.9 × 10^4^]	0.33
RBP_4	1.2 × 10^8^ (0.5 × 10^8^ to 2.4 × 10^8^) [3.1 × 10^8^—5.6 × 10^8^]	1.1 × 10^8^ (0.7 × 10^8 ^to 2.1 × 10^8^) [1.2 × 10^8^—5.5 × 10^8^]	0.93
sCD40-L	0.0 (0.0 to 8.8 × 10^2^) [0.0—423.2 × 10^2^]	0.0 (0.0 to 0.0) [0.0—763.7 × 10^2^]	0.05
SDF_1α	2.0 × 10^4^ (0.0 to 4.2 × 10^4^) [0.0—38.3 × 10^4^]	1.0 × 10^4^ (0.0 to 2.7 × 10^4^) [0.0—26.9 × 10^4^]	**0.02**
MRP_14	4.9 × 10^4^ (2.9 × 10^4^ to 7.9 × 10^4^) [0.0—19.3 × 10^4^]	3.2 × 10^4^ (1.7 × 10^4^ to 5.8 × 10^4^) [0.0—16.8 × 10^4^]	**0.03**
TIMP_1	3.0 × 10^6^ (1.6 × 10^6^ to 4.9 × 10^6^) [0.8 × 10^6^—34.2 × 10^6^]	2.5 × 10^6^ (1.4 × 10^6^ to 3.7 × 10^6^) [0.4 × 10^6^—12.5 × 10^6^]	0.12
TRAIL	0.0 (0.0 to 4.3 × 10^3^) [0.0—23.5 × 10^3^]	0.0 (0.0 to 6.0 × 10^3^) [0.0—49.6 × 10^3^]	0.77

*RBC* red blood cells, *IQR* interquartile range, *pg* pictogram, *PaO*_*2*_ partial pressure of arterial oxygen, *FiO*_*2*_ fraction of inspired oxygen, *CD* cluster of differentiation, *HMGB_1* high-mobility group box 1, *HSP_70* heat shock protein_70, *IDO* indoleamine 2,3-dioxygenase, *IL* interleukin; *PD-L2* programmed cell death ligand 2, *RANTES* regulated upon activation cell expressed and secreted; *RBP_4* retinol-binding protein_4, *sCD* soluble cluster of differentiation, *SDF_1α* stromal cell-derived factor_1 alpha, *MRP_14* myeloid-related protein _14, *TIMP_1* tissue inhibitor matrix metalloproteinase_1, *TRAIL* tumor necrosis factor related apoptosis inducing ligand

### Correlation between SDF_1α and MRP_14 concentrations in SN-RBC and NK cell phenotype and function

**Fig. 3:**
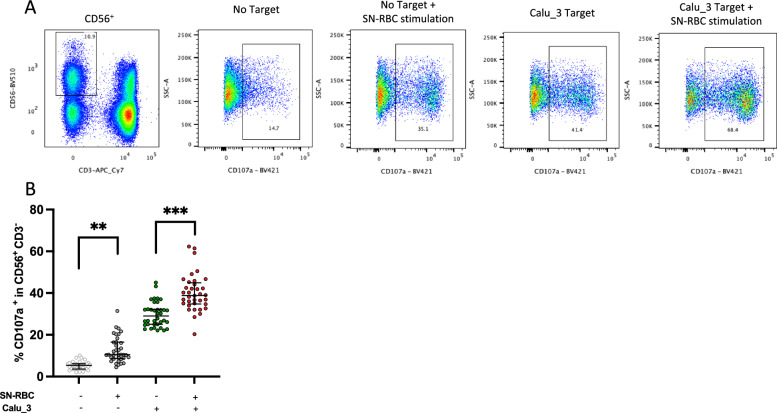
Cytotoxic of NK cells to Calu-3 cell line. PBMC were co-cultured with Calu_3 cell line ("Calu-3 Target") or without Calu_3 (**"**No Target**"**), with or without SN-RBC stimulation. **A** Representative density plots illustrating BV-421 CD107a expression in NK cells (CD3^−^ CD56^+^ in lymphocyte gate) by flow cytometry. **B** Representative histograms of the percentage of CD107a^+^ NK cells in response to Calu_3 stimulation with or without SN-RBC stimulation in flow cytometry (data are shown as the median and interquartile range of 12 SN-RBC tested on 3 distinct healthy donors). **, *P* < 0.01; ***, *P* < 0.001; Calu_3 Target: 5-h coculture between PBMC and Calu_3 cell line (pulmonary epithelial cells) with a Calu_3:NK ratio of 10:1 assuming 10% of NK cells in PBMC; SN-RBC: Red Blood Cell unit supernatant. PBMC, Peripheral Blood Mononuclear Cells

CPB is often described as the "first hit" of TRALI physiopathology because it generates ischemia–reperfusion of the lung. During ischemia–reperfusion, NK cells mature, are recruited to the lungs, and increase lung damage by killing endothelial and epithelial cells expressing stress signals [23–25]. NK cells are innate lymphocytes that constitute up to 15% of resident lung lymphocytes. We hypothesized that NK cell stimulation by inflammatory proteins of RBC could increase lung damage and lead to TRALI ("second hit"). To address this question and more specifically analyze the possible role of SDF_1α and MRP_14 in non-antibody-mediated cases of TRALI, we first analyzed the modification of NK cell receptor expression (Nkp46, Nkp30, DNAM_1, NKG2D, NKG2A, KIR_2D) after stimulation with SN-RBC in vitro. There was no correlation between SDF_1α or MRP_14 concentration in SN-RBC and the change in receptor expression after stimulation compared with the unstimulated condition (Table 3). We then analyzed whether SDF_1α or MRP_14 concentration in SN-RBC correlated with the change in NK cell cytotoxicity to lung epithelial cells (Calu_3 cell line) after stimulation. Four conditions were studied: 1) PBMC alone, 2) PBMC stimulated with SN-RBC, 3) PBMC cocultured with Calu_3 targets, and 4) PBMC stimulated with SN-RBC and cocultured with Calu_3 targets. After SN-RBC stimulation, SDF_1α concentration, but not MRP_14 concentration, correlated with the change in NK cell cytotoxicity to Calu_3 cells (Spearman correlation coefficient r = 0.76, *P* = 0.01 for SDF_1α and r = 0.24, *P* = 0.42 for MRP_14, Table 3 and Fig. 3). This correlation was not confirmed in the HK_2 cell line, suggesting that the change in NK cell cytotoxic response according to SDF_1α concentration in SN-RBC was specific to the lung epithelial cell line.

**Table 3:** Correlation between SDF_1α and MRP_14 concentrations in SN-RBC and the change of NK cell receptor expression and NK cell cytotoxicity

	Correlation with SDF_1α concentrations	*P*	Correlation with MRP_14 concentrations	*P*
Effect on NK cell receptor expression				
NKG2D population MFI	− 0.13	0.52	− 0.13	0.52
NKp46 population MFI	− 0.35	0.09	0.23	0.28
DNAM_1 population MFI	− 0.21	0.31	0.31	0.13
NKp30 MFI	0.37	0.06	0.10	0.63
NKG2A MFI	− 0.35	0.19	− 0.25	0.66
KIR2D^+^				
2DL2/L3/S2^+^ and 2DL1/S1^−^	0.52	0.07	0.14	0.49
2DL2/L3/S2^+^ and 2DL1/S1^+^	0.27	0.09	0.19	0.60
2DL2/L3/S2^−^ and 2DL1/S1^+^	0.41	0.14	0.29	0.61
KIR2D ^–^	− 0.22	0.48	− 0.20	0.51
Effect on NK cell cytotoxic activity				
No target (All subsets)	0.41	**0.03**	0.10	0.63
Against Human airway epithelial cells (Calu_3)	0.76	**0.01**	0.24	0.42
Against Human kidney tubular cells (HK_2)	0.12	0.70	0.31	0.27

This table shows the correlation between the concentration of SDF_1α and MRP_14 in SN-RBC and the changes in NK cell receptor expression (PBMC stimulated by SN-RBC without Calu_3 or HK_2 targets) and NK cell cytotoxicity (PBMC stimulated by SN-RBC with Calu_3 or HK_2 targets). Spearman correlation coefficient: Correlation between SDF_1α concentration in 1 SN-PBMC and the percentage of increase in the expression of NK cell surface receptor or NK cytotoxicity (CD107a expression) after PBMC stimulation with this SN-RBC (compared with non-stimulated condition). The results are representative of 12 distinct SN-RBC tested on PBMC from 3 healthy donors. For each SN-RBC tested, the mean increase in receptor expression or cytotoxicity among the 3 healthy donors was considered. *NK* Natural Killer, *SN-RBC* Supernatant of red blood cell, *PBMC* Peripheral Blood Mononuclear Cells, *KIR* Killer-cell Immunoglobulin-like Receptor, *SDF_1α* Stromal cell-derived Factor_1 alpha, *MRP_14* Myeloïd-related protein 14

### CD56^dim^ NKG2A^+^ NK cells are the main subset involved in the cytotoxic response to the Calu_3 cell line, and the magnitude of the cytotoxic response after stimulation correlates with SDF_1α concentration in SN-RBC

**Fig. 4:**
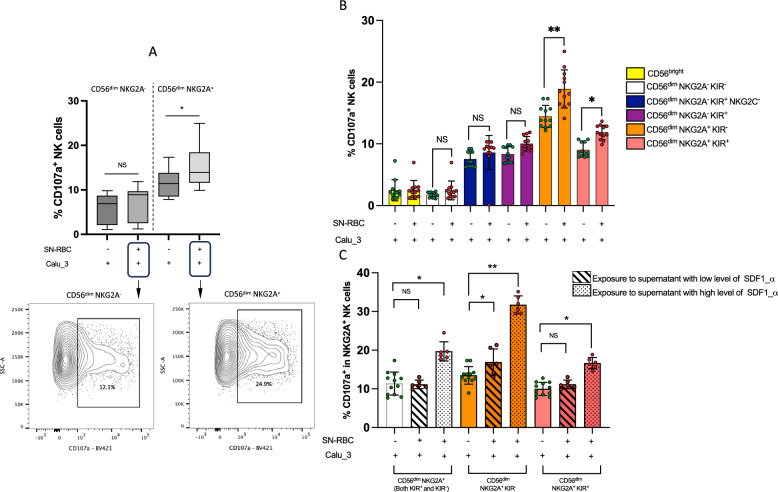
Cytotoxic response of NKG2A^+^ and NKG2A^−^ NK cell subsets. After PBMC culture with or without Calu_3 targets and with or without SN-RBC stimulation, the cytotoxic activity (CD107a-BV421) of several NK cell subsets was analyzed. **A** Box and Whiskers (upper part) showing CD107a-BV421 cytotoxic activity of NK cells according to NKG2A^+^ or NKG2A^−^ expression in response to Calu_3 targets with or without SN-RBC stimulation and density plot (lower part) representing CD107a-BV421 activity of NKG2A^+^ vs. NKG2A^−^ NK cells in response to Calu_3 targets. **B** Representative histograms of CD107a-BV421 cytotoxic activity according to NK cell subset (CD56^bright^, CD56^dim^, NKG2A, and KIR) in response to Calu_3 targets with or without SN-RBC stimulation. **C** Representative histograms of CD107a-BV421 activity in response to Calu_3 targets after stimulation with SN-RBC with low or high levels of SDF_1α. High and low levels were defined as a concentration above or below the median concentration of the 12 SN-RBC used for the experiments. NK cells were analyzed in the lymphocyte gate by flow cytometry after APC Cy7-CD3 BV510-CD56 eFluor780 staining. **, *P* < 0.01; *, *P* < 0.05; **NS**: Non-significant difference; *Calu_3* 5-h coculture between PBMC and Calu_3 cell line (pulmonary epithelial cells) with a Calu_3:NK ratio of 10:1 assuming 10% of NK cells in PBMC; SN-RBC: Red Blood Cell unit supernatant; KIR, Killer Immunoglobulin-like Receptor. PBMC, Peripheral Blood Mononuclear Cells; SDF_1α, Stromal cell-derived Factor_1 alpha. Data are shown as the mean and standard deviation of the effect of 12 SN-RBC tested on 3 different healthy donors. In Panels **B** and **C**, each point represents the mean effect of 1 SN-RBC tested on 3 healthy donors (12 distinct SN-RBC tested)

SDF_1α is a major homeostatic chemokine implicated in several diseases including cancer and acute lung injury [26, 27]. This chemokine has two receptors: CXCR4 and CXCR7. Recently, NK cells were reported to express CXCR4, the expression level of which varies depending on the subset (i.e., in descending order of expression: CD56^dim^ NKG2A^+^ KIR^−^,CD56^dim^ NKG2A^+^ KIR^−^, CD56^dim^ NKG2A^−^ KIR^+^, CD56^dim^ NKG2A^−^ KIR^+^ NKG2C^−^, and CD56^bright^) [28]. Given the possible effect of SDF_1α on NK cell cytotoxicity, we investigated the effect of SN-RBC stimulation on NK cell cytotoxicity against Calu_3 cells according to the NK cell subset in our model. Compared with the CD56^dim^ NKG2A^−^ subset, SN-RBC stimulation significantly increased the cytotoxic response of the CD56^dim^ NKG2A^+^ subset (Fig. 4A). The latter subset was reported to consistently express the highest level of CXCR4 [28]. Looking deeper into each subset, SN-RBC significantly increased the cytotoxicity of both CD56^dim^ NKG2A^+^ KIR^+^ and CD56^dim^ NKG2A^+^ KIR^−^ NK cell subsets (Fig. 4B). To establish a parallel between the results in vitro and the higher exposure to SDF_1α in patients with PaO_2_/FiO_2_ ≤ 300 mmHg, we analyzed flow cytometry results according to SDF_1α concentration in each SN-RBC. Each SN-RBC used for in vitro stimulation was classified as "low-level" or "high-level" if SDF_1α concentration was below or above the median concentration of the 12 SN-RBC randomly selected for the in vitro assay. The increase in NK cell cytotoxicity was more significant after stimulation with high-level SN-RBCs than with low-level SN-RBC. This increase was observed in both CD56^dim^ NKG2A^+^ KIR^−^ and CD56^dim^ NKG2A^+^ KIR^+^ subsets, but was greater in the KIR^−^ subset (Fig. 4C).

### NKG2D is involved in NK cell cytotoxicity to lung epithelial cells

**Fig. 5:**
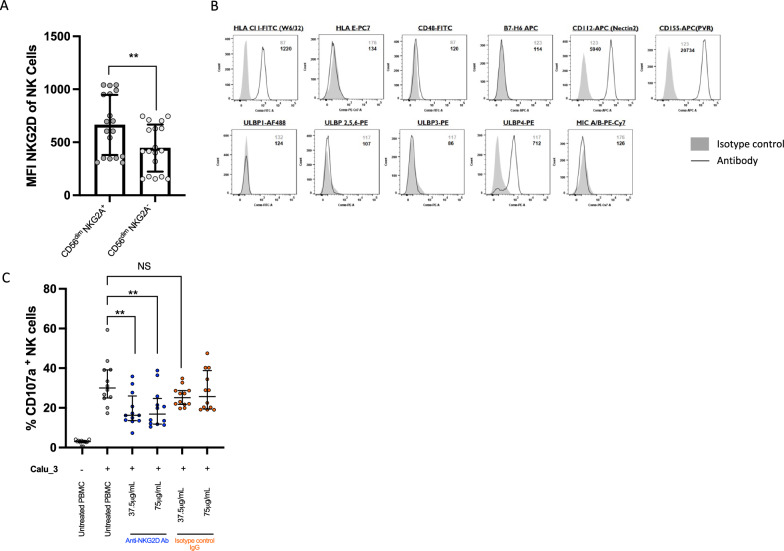
NKG2D-activating receptor is involved in NK cell NKG2A^+^ cytotoxic response to Calu_3 cell line. **A** Representative histograms of NKG2D mean fluorescence intensity (MFI) on NK cells according to the expression of NKG2A receptor (6 conditions for each of the 3 healthy donors, without SN-RBC or Calu_3 targets). Data are shown as the mean and standard deviation. **B** Histogram profiles for different ligands, activating or inhibiting NK cells, expressed on the Calu_3 cell line: ULPB1 to ULPB6 and MICA/B (ligands of NKG2D), B7-H6 (ligand of NKp30), CD48 (ligand of 2B4), and CD112/CD155 (ligands of DNAM-1). Isotype-matched negative controls are shown as gray-filled curves and ligands as white-filled curves. **C** Representative histograms of CD107a activity in sorted NK cells in response to the Calu_3 cell line with or without anti-NKG2D blocking antibody or its isotype (4 distinct wells with sorted NK cells from each of the 3 healthy donors). Data are presented as median and interquartile range. NK cells were analyzed in the lymphocyte gate by flow cytometry after APC Cy7-CD3 BV510-CD56 eFluor780 staining. **, *P* < 0.01; NS: Non-significant difference; Calu_3: 5-h exposition to Calu_3 cell line (pulmonary epithelial cells) with a Calu_3/NK ratio of 10:1 in order to keep the same ratio as in PBMCs and assuming 10% of NK cells in PBMC, PBMC, Peripheral Blood Mononuclear Cells. HLA-Cl I: HLA class I positive cells (complete HLA phenotype is available in supplemental eTable 1). HLA class I is an inhibitory receptor recognized by KIR2DL1, 2DL2, and 2DL3

During ischemia–reperfusion, stress molecules such as NKG2D-ligands are induced on endothelial and epithelial cells [25], leading to NKG2D-dependant lung damage [23, 29, 30]. At least eight ligands of NKG2D are known [31]. Interestingly, NK cell depletion or NKG2D receptor blockade reduced lung injury in the context of ischemia reperfusion [24]. Considering the difference in cytotoxicity to Calu_3 cells with or without SN-RBC stimulation in Fig. 4A, we investigated the differential expression of NKG2D between CD56^dim^ NKG2A^+^ and CD56^dim^ NKG2A^−^ NK cells. We observed that NKG2D expression (expressed in MFI) was higher in the NKG2A^+^ NK cell subset than in the NKG2A^−^ subset (Fig. 5A) without differences in the KIR^−^ vs. KIR^+^ NK cell subset (data not shown). To determine the involvement of NKG2D in NK cell cytotoxicity in our model, we first confirmed the expression of NKG2D-ligands by the Calu-3 cell line (i.e., ULBP_4, Fig. 5B) [32]. Consistently, NKG2D blocking significantly altered NK cell cytotoxicity to the Calu_3 cell line compared with the isotype control (Fig. 5C). These results provide further evidence of the possible involvement of the NKG2A^+^ NK cell subset in NKG2D-dependant lung injury during ischemia–reperfusion.

### NKG2A⁺ NK cells exhibit a cytotoxic and migratory transcriptional program associated with CXCR4 MAPK pathway regulation

**Fig. 6:**
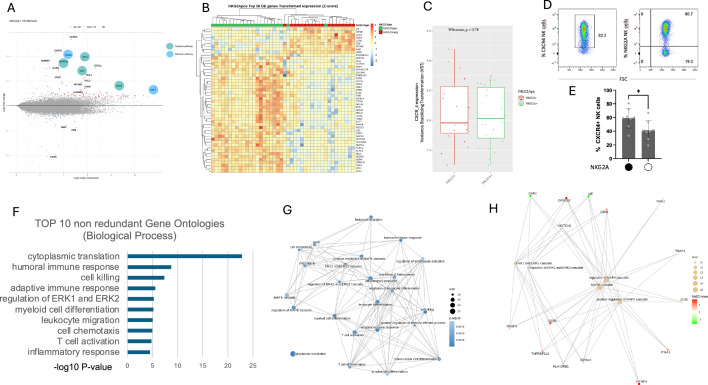
Transcriptomic profiling and CXCR4 expression of NKG2A⁺ and NKG2A⁻ subsets. A MA plot displaying mean normalized expression (log₁₀ scale, x-axis) versus log₂ fold-change between NKG2A⁺ and NKG2A⁻ NK cells. Each point represents a gene; genes meeting the adjusted significance threshold (FDR < 0.01) are highlighted in color. **B** Heatmap of the top 50 differentially expressed genes ranked by adjusted *p*-value. Rows correspond to genes and columns to individual samples. Expression values are scaled by row (Z-score). Genes such as *SELL*, *GZMK*, *CD2*, *GNLY*, and *IFNG* are among those most enriched in the NKG2A⁺ subset. **C** Differential mRNA expression between NKG2A^+^ and NKG2A^−^ subsets for Granzyme B, CXCR4 and perforine in the RNA-sequencing analysis (calculated by Variance stabilizing transformation based on delta method). Variance transformed. **D** Representative flow cytometry plots showing gating strategy and CXCR4^+^ frequency of NKG2A^+^ and NKG2A^−^ cell subsets. CXCR4 expression was documented using PE-CXCR4-specific mAb. **E** Bar graphs comparing the frequency of CXCR4 in the NKG2A^+^ and NKG2A.^−^ cell subsets (n = 8). Comparisons were made by Mann–Whitney tests. **F** Gene Ontology (GO) enrichment analysis of upregulated genes in the NKG2A⁺ subset. Bars indicate the top 10 non-redundant biological processes with adjusted *p* < 0.05, ranked by –log₁₀ (*p-*value).A complete list of enriched terms is provided in Supplemental #2. **G** Network representation of significantly enriched GO terms based on semantic similarity clustering. Nodes represent biological processes; edges reflect shared gene overlap. Major clusters correspond to translation, cytotoxicity, migration, and ERK-related signaling. Node size is proportional to the number of contributing genes; color intensity corresponds to significance (FDR value). **H** Focused enrichment map showing genes contributing to the “MAPK cascade” and “regulation of ERK1 and ERK2 signaling” GO terms. Differentially expressed genes include *DUSP2*, *DUSP4*, *EPHA4*, *PIK3R2*, and *GSTP1*. Color scale indicates relative expression (log₂ fold-change) between NKG2A⁺ and NKG2A⁻ subsets expression of CXCR4 showing no significant differences between NKG2A + and NKG2A- samples (wilcoxon test: p-value = 0.78

To bring further evidence on the possible role of NKG2A^+^ NK cells in organ damage after interstitial migration in the physiopatholgy of TRALI, we reanalyzed our previously published RNA-seq dataset comparing NKG2A⁺ and NKG2A⁻ NK cell subsets [33]. We found 127 genes differentially expressed between the 2 subsets. Compared with NKG2A^−^ subset, NKG2A^+^ cell subsets showed 102 upregulated and 25 downregulated genes. The MA plot (Fig. 6A) illustrates the global transcriptional distribution between the two populations and highlights a cluster of genes upregulated in NKG2A⁺ NK cells, including SELL, GZMK, CD2, GPR183, and GNLY which are classically associated with adhesion and cytotoxicity. Unsupervised hierarchical clustering of the top 50 differentially expressed genes further confirmed that NKG2A⁺ NK cells display a distinct transcriptional identity enriched for effector molecules and adhesion markers (Fig. 6B). The transcriptomic expression of CXCR4 was not significantly different between NKG2A^+^ and NKG2A^−^ subset (Fig. 6C), however cytometry analysis confirmed the higher percentage of NKG2A^+^ NK cells among CXCR4 positive population, which corroborate the higher cytotoxic activity of NKG2A^+^ subset after stimulation with SN-RBC with high SDF_1α concentration (Fig. 6D, E). Gene Ontology (GO) enrichment analysis of upregulated genes identified biological processes linked to cytoplasmic translation, humoral and adaptive immune responses, cell killing, regulation of ERK1 and ERK2 signaling, and leukocyte migration (Fig. 6F and see Supplemental#2 for the complete GO list). Network-based visualization of enriched terms revealed strong interconnections between cytotoxicity, cell migration, and ERK pathway regulation, underscoring the coexistence of effector activation and migratory potential within the NKG2A⁺ subset (Fig. 6G), and that the MAPK/ERK pathway signaling under CXCR4 was enriched. The RNAseq analysis confirms therefore that NKG2A^+^ NK cells exhibit a genetic background prone to fix onto the endothelium, to migrate and to exert cytotoxicity when reaching lung interstitium. Given the established role of the SDF_1α/CXCR4 axis in controlling NK-cell trafficking and tissue retention, we next examined its transcriptional and signaling context in NKG2A⁺ NK cells. Although CXCR4 mRNA expression did not differ significantly between NKG2A⁺ and NKG2A⁻ subsets, pathway enrichment analysis revealed differential regulation of genes associated with the MAPK/ERK cascade, a key pathway downstream of CXCR4 activation [34]. Specifically, the focused enrichment map identified DUSP2, DUSP4, EPHA4, PIK3R2, and GSTP1 as regulatory components modulated in the NKG2A⁺ subset (Fig. 6H). This transcriptional pattern suggests that, despite unchanged CXCR4 transcript levels, NKG2A⁺ NK cells possess a MAPK/ERK regulatory profile capable of fine-tuning responsiveness to SDF_1α stimulation. Together, these data indicate that NKG2A⁺ NK cells exhibit a transcriptional program coupling cytotoxic activation with CXCR4-dependent signaling. The modulation of MAPK/ERK regulators suggests that these cells are transcriptionally configured for context-dependent activation of CXCR4-mediated pathways, enabling endothelial adhesion, interstitial migration, and cytotoxic effector function within the lung microenvironment during TRALI.

Interestingly, based on RNAseq analysis, NKG2A^+^ subset shares phenotype characteristic of NK2 and NKint subsets with the expression of soluble factors that modulate immune responses (XCL1 and XCL2), molecules implicated in cell migration and tissue homing (CD44 and SELL) and Granzyme k (GZMK) as described previously described [35].

### Characteristics of blood donor and concentration of SDF_1α

**Fig. 7:**
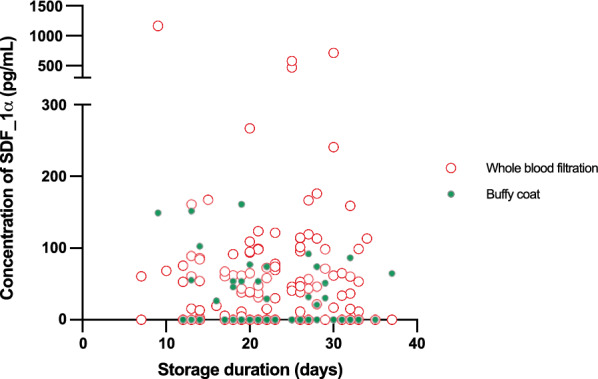
Effect of blood storage duration on SDF_1α concentration in SN-RBC. SDF_1α concentration according to the storage duration of the red blood cell units at the blood bank and the preparation method of the blood donation (Whole blood filtration vs. Buffy coat). The Pearson correlation coefficient between storage duration and the concentration of SDF_1α was r = -0.170, *P* = 0.11 after Buffy coat and r = -0.039, *P* = 0.67 after whole blood filtration (each point stands for one of the 204 SN-RBC tested by Luminex immune assay). During "whole blood filtration" the unseparated components (whole blood of the donor) undergo in-line leukocyte filtration, followed by centrifugation to separate plasma and red blood cells. For the "Buffy coat" preparation, whole blood undergoes centrifugation to separate red blood cells, platelets, and plasma. Leukocytes and platelets are then removed, and red blood cells are filtered. SN-RBC, supernatant of red blood cell unit; SDF_1α, Stromal cell-derived Factor_1 alpha

Considering that SDF_1α from RBC appears to be associated with ALI after transfusion in cardiac surgery patients and that exposure of PBMC to SN-RBC with high levels of SDF_1α enhanced damage to the lung epithelial cell line, we investigated whether the SDF_1α concentration could be determined based on RBC characteristics (Table 4). We found that RBC contained higher levels of SDF_1α when the donor was a woman (*P* < 0.01), or had a platelet count > 300 × 10^9^/L (*P* < 0.01), or when the blood donation was prepared by "whole blood filtration" compared with "buffy coat" (*P* < 0.01). No difference in SDF_1α concentration was observed depending on the blood group, rhesus factor, or history of pregnancy. Blood storage duration, which appears to have no impact on patient outcomes [36], was not associated with SDF_1α concentrations (Fig. 7).

**Table 4:** Comparison of mean concentrations of SDF_1α in Red Blood Cell Unit supernatant depending on donor characteristics and filtration technique

	N	SDF_1α concentrations (pg/ml) mean (± SD)	*P*-value
Gender			
Male	102	23.23 (± 48.31)	** < 0.01**
Female	102	76.48 (± 150.52)	
History of pregnancy			
Yes	70	88.74 (± 178.07)	0.62
No	32	49.66 (± 46.42)	
Age (years)			
< 50	131	52.55 (± 120.43)	0.24
> 50	73	44.64 (± 104.81)	
Blood group			
A	97	36.52 (± 50.96)	0.86
B	11	33.10 (± 34.42)	
AB	5	21.96 (± 26.50)	
O	91	67.63 (± 161.65)	
Rhesus			
Positive	166	53.01 (± 121.14)	0.29
Negative	38	36.06 (± 80.26)	
Platelet count			
< 300 × 10^9^/L	156	43.34 (± 116.31)	** < 0.01**
> 300 × 10^9^/L	48	71.02 (± 108.72)	
Preparation methods			
Buffy coat	87	17.22 (± 36.48)	** < 0.01**
Whole blood filtration	117	73.65 (± 143.76)	

Composition of 204 SN-RBC unit analyzed by multiplex immune-assay. *SDF_1α* Stromal cell-derived Factor_1 alpha; *SD* standard deviation; *pg* picogram; *mL* milliliter; *RBC* Red Blood Cells. During "whole blood filtration", the unseparated components (whole blood of the donor) undergo in-line leukocyte filtration, followed by centrifugation to separate plasma and red blood cells. For the "Buffy coat" preparation, whole blood undergoes centrifugation to separate red blood cells, platelets, and plasma. Leukocytes and platelets are then removed, and red blood cells are filtered

## Discussion

Patients who developed postoperative hypoxemia had higher transfusion-related exposure to SDF_1α. All participants received transfusions but only 33.5% developed ALI, suggesting that TRALI requires both a specific patient status and a specific composition of RBC. This study uncovers a possible new immunopathology of non-antibody-mediated TRALI in cardiac surgery patients, which could be described as follows: 1) ischemia–reperfusion increases NKG2D ligands on lung epithelial cells [25], and 2) an increase in NKG2D-dependent cytotoxicity of NKG2A^+^ NK cells after transfusion of RBC containing high levels of stimulating cytokines, including SDF_1α. However, we did not establish a causal link between SDF_1α exposure and the onset of ALI after cardiac surgery.

Little is known about the role of NK cells in the immunopathology of TRALI. Recently, circulating NK cells were reported to express CXCR4 which is also ubiquitously expressed on leukocytes, bone marrow hematopoietic stem cells, as well as epithelial, endothelial, and stromal cells from the lung [37]. SDF_1α/CXCR4 interaction usually promotes progenitor cell retention in the bone marrow niche. During ischemia, the latter interaction is disrupted, and leukocytes are recruited to the injured organs [38]. Activation of the SDF_1α/CXCR4 axis in the plasma [39], lung tissue, and bronchoalveolar lavage fluid has been reported to play a key role in the early development of ALI after CPB [40]. CXCR4 antagonization has been reported to mitigate acute respiratory distress syndrome [41], and reduce leukocyte influx in the kidney [42], after ischemia–reperfusion. The present study provides further evidence on the possible importance of SDF_1α/CXCR4 axis and NKG2D/NKG2D-ligand interaction in ALI during ischemia–reperfusion [30].

Consistent with this concept, transcriptomic profiling of sorted NKG2A^+^ NK cells revealed enrichment of genes involved in cytotoxic effector function, adhesion, and leukocyte migration, indicating that this subset is transcriptionally equipped to adhere to activated endothelium, traffic into the lung interstitium, and injure epithelial targets. In addition, the regulation of MAPK ERK pathway components within the CXCR4 signaling context suggests that NKG2A^+^ NK cells are transcriptionally primed for context dependent integration of CXCL12 signals during ischemia reperfusion and transfusion exposure, thereby linking their cytotoxic potential to the SDF_1α/CXCR4 axis in TRALI.

TRALI was mainly described after plasma transfusion but it can be triggered by any plasma-containing blood product and can occur up to 72 h after transfusion [43]. Despite the small amount of plasmatic protein, RBC transfusion is associated with the largest number of reported cases of TRALI [14]. This strengthens the notion that RBC composition should be monitored to better stratify the risk of transfusion-related organ dysfunction. Previous studies have reported that aged red blood cells contain more proinflammatory mediators or bioactive lipids [16, 44]. Our data suggest that there is no link between SDF_1α composition and RBC storage duration. Among peripheral blood components, platelets are thought to be the main source of SDF_1α.[45] Accordingly, RBC from donors with platelet counts > 300 × 10^9^/L or prepared by whole blood filtration (i.e., reported to contain more platelet-derived extracellular vesicles) [46], showed the highest concentration of SDF_1α.

Several limitations should be discussed. First, our experiments do not establish a causal link between high SDF_1α exposure and the onset of ALI after cardiac surgery. However, establishing such a link is elusive given the multiple cofactors involved in lung dysfunction in cardiac surgery patients and the overlap among several entities, such as ischemia–reperfusion, infection/aspiration, overload, and lesional edema, regardless of transfusion. In the same vein, the ROC curve analysis assessing the ability of SDF_1α concentration to predict the onset of postoperative ALI yielded limited results (AUC 0.64, 95% CI [0.53 to 0.75], see supplemental eFigure 1). Nevertheless, our study suggests that SDF_1α concentration in RBC supernatant may be associated with the onset of acute lung injury after cardiac surgery. Moreover, these results support better characterization of the determinants of RBC composition and the development of new immune strategies to mitigate transfusion side effects. Second, the use of Calu_3 cells to study NK cell cytotoxicity toward the lung epithelium during TRALI is questionable. However, Calu_3 cells have recently been used to study the human lung secretome [47] and are known to form tight junctions, desmosomes, and microvilli, mimicking the lung epithelium. Although a cell line cannot reproduce a two-hit model, we selected a cell line expressing stress molecules such as NKG2D ligand, as lung tissue does after ischemia–reperfusion [23]. Third, we did not establish a causal link between SDF_1α exposure and the enhancement of NKG2A^+^ NK cell cytotoxicity or the onset of ALI after cardiac surgery. We showed that NK cell cytotoxicity increased after exposure to SN-RBC with high concentrations of SDF_1α, presumably containing other inflammatory proteins. We acknowledge that selecting a short list of proteins for the multiplex immune assay, rather than using a proteomic approach, could have biased the analyses. Fourth, many confounding factors can lead to over- or under-diagnosis of TRALI after cardiac surgery. Consequently, the classification of participants into the ALI or non-ALI group did not follow the strict definition of TRALI. Notably, some participants developed hospital-acquired pneumonia, but mainly after day 3 postoperatively. Similarly, the diagnosis of fluid overload is difficult given the equivocal results of left atrial pressure measurement postoperatively. Fifth, 24-h SDF_1α stimulation of sorted NK cells was previously reported to decrease cytotoxicity to K562 cells [48]. This suggests that the microenvironment (i.e., cytokines and cells) and the timing of stimulation are involved in the increase in NK cell cytotoxicity toward lung epithelial cells after SN-RBC exposure. Sixth, although RNA sequencing strengthened the hypothesis regarding the cytotoxic role of NKG2A^+^, transcriptomic analyses of CXCR4 in NKG2A^+^ vs. NKG2A^−^ subsets revealed additional complexity in the pathophysiology of TRALI. Differences in cytotoxicity and responsiveness to SDF_1α between the 2 subsets may be due to differential expression of CXCR4 and to genetic background. Moreover, important interindividual heterogeneity of CXCR4 function may explain differential susceptibility to developing TRALI in the population.

## Conclusion

Our data suggest that high exposure to SDF_1α through transfusion may be associated with the onset of ALI after cardiac surgery. In vitro, SN-RBC with high levels of SDF_1α increased NKG2D-dependent NK cell cytotoxicity. We also found that RBC from donors with platelet counts > 300 × 10^9^/L or prepared by whole blood filtration had the highest SDF_1α levels, as did RBC from female donors. Modulation of the SDF_1α/CXCR4 axis appears to be a promising target for reducing organ dysfunction after ischemia–reperfusion and mitigating the risk of ALI.

### Clinical perspective

#### Competency in patient care and procedural skills

The risk of transfusion-related acute lung injury after cardiopulmonary bypass could depend on the inflammatory contents of packed red blood cells received during surgery.

#### Translational outlook

The characterization and the close monitoring of the composition of blood products may prove beneficial to reducing transfusion-related organ dysfunction and improving the safety of transfusion.

## Supplementary Information

Additional file 1.

Additional file 2.

## Data Availability

The datasets used and/or analysed during the current study are available from the corresponding author on reasonable request.

## Abbreviations

ALI: Acute lung injury
ARDS: Acute respiratory distress syndrome
CPB: Cardiopulmonary bypass
CXCR4: C-X-C motif chemokine receptor_4
EFS-PL: Etablissement français du sang des pays de Loire
FFP: Fresh Frozen Plasma
FiO_2_: Fraction of inspired oxygen
HLA: Human leukocyte antigen
ICU: Intensive care unit
NK: Natural killer
PBMC: Peripheral blood mononuclear cell
PaO_2_: Partial pressure of oxygen
RBC: Red blood cell unit
SN-RBC: Supernatant of Red blood cell unit
SDF_1α: Stromal cell-derived factor_1 alpha
TACO: Transfusion associated circulatory overload
TRALI: Transfusion-related acute lung injury
