# Supplementary material for: The role of SDF_1α/CXCR4 axis in non-antibody mediated TRALI in cardiac surgery patients
Source: Intensive Care Med Exp. 2026 Jul 23;14:100. doi: 10.1186/s40635-026-00919-z (PMC13396328; doi:10.1186/s40635-026-00919-z)
Supplement: Supplementary file 2 — Additional file 2. [file 40635_2026_919_MOESM2_ESM.docx]

Supporting Information Text

**eMethods 1**

RNA-seq data generation and primary processing were performed as previously described in the original study from which these data were obtained.^1^ Briefly, 3’ digital gene expression profiling was carried out on total RNA using a template-switching protocol with sample barcodes and unique molecular identifiers, followed by pooling, amplification, library preparation, and sequencing on a NovaSeq 6000 system, as described previously.^2^ Reads were trimmed and aligned to the hg38 reference genome, and UMI-based gene-level counts were derived from the multimapping matrix to preserve signal for highly homologous KIR genes. Differential expression between NKG2A-positive and NKG2A-negative NK cell subsets was assessed with DESeq2, considering genes with non-zero counts and declaring significance for an adjusted P-value below 0.05 and a nominal P-value below 0.01. Gene set enrichment analysis of ranked gene lists was performed with the fgsea package.

**eTable 1 – Phenotype of Calu-3 and HK_2 cell line for NK cell ligands**

|  | **Calu_3 Cell line** | **HK_2 Cell line** |
| --- | --- | --- |
| HLA-haplotypes | HLA_A 24/68, HLA_B 07/51, HLA_C07 (C1)/C15 (C2) | HLA_A 02/30, HLA_B 13/44, HLA_C02 (C2)/C06 (C2) |
| Positive markers (expressed) | ULBP_4, CD112, CD155 | ULBP_2, 5, 6, MICA/B, CD112, CD155, CD274. |
| Negative markers (not expressed) | HLA_E , CD48, ULBP_1, 2, 3, 5, 6, MICA/B, B7/H6, CD274 | HLA_E, CD48, ULBP_3, ULBP_4, B7/H6, |

**Legend eTable 1:** Killer-cell Immunoglobulin-like receptor (**KIR**) can be activating or inhibitory receptors.^3^ KIR2DL1 and KIR2DS1 can recognize C1, with an inhibitory (2DL1) or activatory (2DS1) activity, whereas KIR2DL2, 2DL3 and 2DS2 recognize class C2 molecules

|  | Low SDF_1α exposure  (Below the median exposure of the participants)  **N = 112** | High SDF_1α exposure  (Over the median exposure of the participants)  **N = 49** | *P*-value |
| --- | --- | --- | --- |
| Mean time on Norepinephrine support, hours (±SD) | 29.3 (±64.2) | 26.5 (±42.5) | 0.71 |
| Mean time on Dobutamine support, hours (±SD) | 17.2 (±38.7) | 23.0 (±36.5) | 0.62 |
| Mean time on intravenous insulin, hours (±SD) | 35.3 (±41.6) | 54.4 (±98.3) | 0.38 |
| Highest lactate level, mmol/L, median [IQR] | 1.9 [1.5 to 2.2] | 1.7 [1.4 to 2.3] | 0.56 |
| Hospital acquired infection, n° (%) | 6 (5.4%) | 5 (10.2%) | 0.31 |
| PaO_2_/FiO_2_ ratio ≤ 200 mmHg in the first 3 days, n (%) | 12 (10.7%) | 26 (53.1%) | **< 0.01** |
| Acute kidney injury in the first 48 hours, n° (%) | 5 (4.5%) | 12 (24.5%) | **< 0.01** |
| Dialysis, n° (%) | 2 (1.8%) | 1 (2.0%) | 0.99 |
| Mean duration of mechanical ventilation, hours (±SD) | 19.4 (±55.4) | 27.1 (±70.3) | 0.41 |
| Length of ICU stay, median [IQR], days | 1.0 [1.0 to 2.0] | 1.0 [1.0 to 4.0] | 0.10 |
| Length of hospital stay, median [IQR], days | 14.0 [9.0 to 20.0] | 14.0 [9.0 to 23.0] | 0.46 |
| Deaths, n° (%) | 3 (2.7%) | 1 (2.0%) | 0.99 |
| Postoperative extracorporeal life support, n° (%) | 1 (0.9%) | 1 (2.0%) | 0.52 |
| Diagnosis of cancer in the following year, n° (%) | 2 (1.8%) | 0 | - |

**eTable 2: Supplemental data for postoperative outcome**

**Legend eTable 2**: **SDF_1:** Stromal cell-derived Factor_1 alpha; **SD**: Standard Deviation; **ICU**: Intensive Care Unit; **pg**: picogram; **IQR**: Interquartile range. **PaO_2_**: partial pressure of arterial oxygen; **FiO_2_**: fraction of inspired oxygen. Acute Kidney Injury was defined at 48 hours postoperatively as a decrease of at least 25% in the estimated glomerular filtration rate (according to the Modification of Diet in Renal Disease equation) or an increase in creatinine of at least 1.5-fold compared with the preoperative period.^4^

**eFigure 1: SDF-1α best threshold determination by the ROC curve approach**

**Legend for eFigure 1:** Area under the curve (AUC) = 0.64; 95% CI [0.53 to 0.75]. The optimal SDF-1 value for predicting the onset of acute lung injury after cardiac surgery was 18 034 (using the Youden method).

1. Ferron E, Jullien M, Braud M, et al.: Molecular Interactions Between NK Cells and Acute Leukemic Cells: KIR2DL5 Drastically Limits NK Cell Responses. J Clin Immunol 2025; 45:118

2. Chaumette T, Cinotti R, Mollé A, et al.: Monocyte Signature Associated with Herpes Simplex Virus Reactivation and Neurological Recovery after Brain Injury. Am J Respir Crit Care Med 2022; 206:295–310

3. Pende D, Falco M, Vitale M, et al.: Killer Ig-Like Receptors (KIRs): Their Role in NK Cell Modulation and Developments Leading to Their Clinical Exploitation. Front Immunol 2019; 10:1179

4. Kuitunen A, Vento A, Suojaranta-Ylinen R, Pettilä V: Acute Renal Failure After Cardiac Surgery: Evaluation of the RIFLE Classification. The Annals of thoracic surgery 2006; 81:542–6
